# Association of low adherence to weekly cisplatin with outcomes in patients with head and neck squamous cell carcinoma: a retrospective cohort study

**DOI:** 10.1186/s12885-024-12615-w

**Published:** 2024-07-13

**Authors:** Jas Virk, Jasmin Gill, Fatemeh Fekrmandi, Austin Iovoli, Mark Farrugia, Ayham Al-Afif, Kimberly Wooten, Vishal Gupta, Ryan McSpadden, Moni A. Kuriakose, Michael R. Markiewicz, Wesley L. Hicks, Sung Jun Ma, Anurag K. Singh

**Affiliations:** 1 Jacobs School of Medicine and Biomedical Sciences, University at Buffalo, The State University of New York, 955 Main Street, Buffalo, NY 14203 USA; 2 University at Buffalo, The State University of New York, 12 Capen Hall, Buffalo, NY 14260 USA; 3 Department of Radiation Medicine, Roswell Park Comprehensive Cancer Center, 665 Elm and Carlton Streets, Buffalo, NY 14203 USA; 4 Department of Head and Neck Surgery, Roswell Park Comprehensive Cancer Center, Elm and Carlton Streets, Buffalo, NY 14203 USA; 5 Department of Oral and Maxillofacial Surgery, School of Dental Medicine, University at Buffalo, The State University of New York, 3435 Main Street, Buffalo, NY 14214 USA; 6 Department of Neurosurgery, Jacobs School of Medicine and Biomedical Sciences, University at Buffalo, The State University of New York, 955 Main Street, Buffalo, NY 14203 USA; 7 Department of Radiation Oncology, The Arthur G. James Cancer Hospital and Richard J. Solove Research Institute, The Ohio State University Comprehensive Cancer Center, 460 W 10 Ave, Columbus, OH 43210 USA

**Keywords:** Adherence, Compliance, Concurrent chemotherapy, ChemoRT, Head and neck cancer, Weekly cisplatin

## Abstract

**Background:**

The National Comprehensive Cancer Network (NCCN) guideline recommends consideration of weekly cisplatin as an alternative option for patients with head and neck cancer undergoing definitive chemoradiation. However, in a recent phase III trial (ConCERT), 20% of patients treated with weekly cisplatin could not receive a total of 200 mg/m^2^, and the association of low adherence to weekly cisplatin and cancer control outcomes remains unclear. To fill this knowledge gap, we performed an observational cohort study of patients with head and neck cancer undergoing definitive chemoradiation with weekly cisplatin.

**Methods:**

Our institutional database was queried for patients with non-metastatic head and neck cancer who underwent definitive chemoradiation with weekly cisplatin (40 mg/m^2^) between November 2007 and April 2023. Adherence to weekly cisplatin was defined as receiving at least 5 cycles with a total cumulative dose of 200 mg/m^2^. Survival outcomes were evaluated using Kaplan–Meier method, log-rank tests, Cox proportional hazard multivariable (MVA) analyses. Logistic MVA was performed to identify variables associated with low adherence to weekly cisplatin. Fine-Gray MVA was performed to analyze failure outcomes with death as a competing event.

**Results:**

Among 119 patients who met our criteria, 51 patients (42.9%) had low adherence to weekly cisplatin. Median follow up was 19.8 months (interquartile range 8.8–65.6). Low adherence to weekly cisplatin was associated with worse overall survival (adjusted hazards ratio [aHR] 2.94, 95% confidence interval [CI] 1.58–5.47, *p* < 0.001) and progression-free survival (aHR 2.32, 95% CI 1.29–4.17, *p* = 0.005). It was also associated with worse distant failure (aHR 4.55, 95% CI 1.19–17.3, *p* = 0.03), but not locoregional failure (aHR 1.61, 95% CI 0.46–5.58, *p* = 0.46). KPS < 90 was the only variable associated with low adherence to weekly cisplatin (adjusted odds ratio [aOR] 2.67, 95% CI 1.10–6.65, *p* = 0.03).

**Conclusion:**

Our study suggested that over 40% of patients underwent fewer than 5 weekly cisplatin cycles and that low adherence to weekly cisplatin was an independent, adverse prognostic factor for worse survival and distant failure outcomes. Those with reduced adherence to weekly cisplatin were more likely to have poor performance status. Further studies are warranted to improve the adherence to chemotherapy and outcomes.

## Introduction

Definitive chemoradiation remains one of the standard of care treatment options for locally advanced head and neck cancer [1]. High-dose cisplatin given every 3 weeks is currently a category 1, preferred concurrent chemotherapy regimen based on the National Comprehensive Cancer Network (NCCN) guideline [1]. However, given its toxicity profiles, nearly a third of patients could not receive the 3rd cycle in the RTOG 0129 trial [2].

Alternatively, the national guideline also recommends low-dose weekly cisplatin as an additional treatment option [1]. In a prior meta-analysis and recently completed ConCERT trial, there was no statistically significant difference in locoregional control and overall survival between high- and low-dose cisplatin [3, 4] as similarly seen in the postoperative setting [5]. Despite such favorable outcomes, adherence to low-dose cisplatin remains challenging. The meta-analysis suggested comparable chemotherapy interruptions for high- versus low-dose cisplatin [3], and up to a third of patients with low-dose cisplatin could not receive a total of 200 mg/m^2^among patients with head and neck cancer, comparable to those with cervical cancer [4, 6–9]. However, the impact of poor adherence to low-dose cisplatin on cancer control outcomes remains unclear. To fill this knowledge gap, we performed an observational cohort study of patients with head and neck cancer undergoing definitive chemoradiation with low-dose cisplatin, evaluating the association between the adherence to low-dose cisplatin and outcomes.

## Methods

This study was approved by Roswell Park Comprehensive Cancer Center institutional review board (EDR 103707) and follows the Strengthening the Reporting of Observational Studies in Epidemiology (STROBE) reporting guideline. A waiver of consent was obtained from the institutional review board given the retrospective nature of our study. Consent process would not be feasible, and obtaining consents in retrospect would pose a greater risk than the waiver.

Our institutional database was queried for patients with non-metastatic head and neck cancer who underwent definitive chemoradiation with weekly cisplatin (40 mg/m^2^) between November 2007 and April 2023. All patients underwent 69.96–70 Gy/33–35 fractions using intensity modulated radiation therapy [10], and treatment volumes were previously described [10] based on several guidelines [11–13]. Of note, in our institutional practice, we routinely covered levels II-V unilaterally for well-lateralized tonsils and bilaterally for other primary oropharyngeal sites, with level Ib included for those with invasion into oral cavity or select level II lymphadenopathy per treating physician’s discretion. All patients underwent baseline positron emission tomography/computed tomography (PET/CT) which guided the staging and contouring. No dose or volume reduction was performed for those with human papillomavirus (HPV)-associated head and neck cancer. Excluded patients were those who underwent surgery, radiation alone, other chemotherapy regimens, or had metastatic cancer.

Supportive care measures for skin, oral mucositis, and pain control have been previously described [14–16]. In our institutional practice, high-dose cisplatin given every 3 weeks was routinely given for those with excellent performance status, normal renal function, and limited medical comorbidities. Otherwise, for other patients with normal renal function who had a reasonable performance status with some medical comorbidities, it was a treating physician’s discretion to give weekly cisplatin, fine-tune its schedule such as delaying or discontinuing certain cycles, and coordinate other necessary infusions as clinically appropriate. For example, given the nature of weekly cisplatin, weekly peripheral blood tests were routinely performed to rule out metabolic abnormalities. Antibiotics and escalated care were performed as clinically necessary in the setting of neutropenic fever or other infectious etiologies.

Adherence to low-dose cisplatin was defined as receiving at least 5 cycles with a total cumulative dose of 200 mg/m^2^. A total of 200 mg/m^2^was chosen as a benchmark since there were no statistical differences in survival between 2 versus 3 high-dose cisplatin cycles in the RTOG 0129 [2]. Other relevant variables included age, gender, smoking history, Karnofsky Performance Status (KPS), race, body mass index (BMI), primary disease site, cancer staging based on the American Joint Committee on Cancer (AJCC) 7th edition, and HPV status. All missing variables were coded as unknown for analysis. Race was self-reported by patients. Non-Caucasian patients were grouped into a single category prior to our analysis given small subgroup sample sizes, and they included African American, American Indian or Alaska Native, Asian, Hispanic, and others who were unknown or declined to answer.

The primary endpoint was overall survival (OS) and progression-free survival (PFS). OS was defined as the time interval from diagnosis to death by any cause or last follow up. PFS was defined similarly as OS, except that PFS included any tumor progression in addition to death by any cause or last follow up. Other endpoints were locoregional failure (LRF) and distant failure (DF), defined as the time intervals from diagnosis to tumor recurrences within and outside head and neck, respectively.

### Statistical analysis

Baseline characteristics were compared using Fisher exact test and Mann–Whitney U tests as appropriate. Survival outcomes were evaluated using Kaplan–Meier method, log-rank tests, Cox proportional hazard multivariable (MVA) analyses. Logistic MVA was performed to identify variables associated with poor adherence to low-dose cisplatin. Fine-Gray MVA was performed to analyze LRF and DF outcomes with death as a competing event. All MVA models were built based on all baseline variables as listed previously.

All *p* values were two-sided, and *p* values less than 0.05 were considered statistically significant. All analyses were performed using R (version 4.2.1, R Project for Statistical Computing, Vienna, Austria).

## Results

A total of 119 patients (101 male [84.9%], median [interquartile range] age, 63.5 [56–71] years) met our criteria (Table 1). The majority patients were former smoker (*n* = 64, 53.8%) with good KPS of 90–100 (*n* = 81, 68.1%) who underwent definitive chemoradiation for oropharyngeal cancer (*n* = 66, 55.5%). Of 119 patients, 51 patients (42.9%) had poor adherence to low-dose cisplatin. When compared between those with or without adherence to low-dose cisplatin, there were more patients with poor KPS and other racial backgrounds (Table 1). Median follow up was 19.8 months (interquartile range 8.8–65.6).

**Table 1:** Baseline characteristics

	> / = 5 Cycles		< 5 Cycles		P
	N	%	N	%	
Gender					1.00
Male	58	85.3	43	84.3	
Female	10	14.7	8	15.7	
Smoker					0.15
Never	21	30.9	9	17.6	
Former	36	52.9	28	54.9	
Current	11	16.2	14	27.5	
Age					0.85
< 65	38	55.9	27	52.9	
65 or older	30	44.1	24	47.1	
KPS					0.003
90–100	54	79.4	27	52.9	
< 90	14	20.6	24	47.1	
Race					0.02
White	63	92.6	39	76.5	
Other	5	7.4	12	23.5	
BMI					0.68
Normal	18	26.5	14	27.5	
Underweight	1	1.5	3	5.9	
Overweight	27	39.7	21	41.2	
Obese	21	30.9	13	25.5	
Not available	1	1.5	0	0.0	
Site					0.17
Oropharynx	41	60.3	25	49.0	
Larynx	12	17.6	16	31.4	
Oral cavity	0	0.0	1	2.0	
Other	15	22.1	9	17.6	
T staging					0.27
1–2	37	54.4	22	43.1	
3–4	31	45.6	29	56.9	
N staging					0.30
0–1	16	23.5	17	33.3	
2–3	52	76.5	34	66.7	
HPV					0.46
Negative	11	16.2	12	23.5	
Positive	36	52.9	22	43.1	
Not available	21	30.9	17	33.3	

*N* Number of patients, *BMI* Body mass index, *KPS* Karnofsky performance status, *HPV* Human papillomavirus

**Fig. 1:**
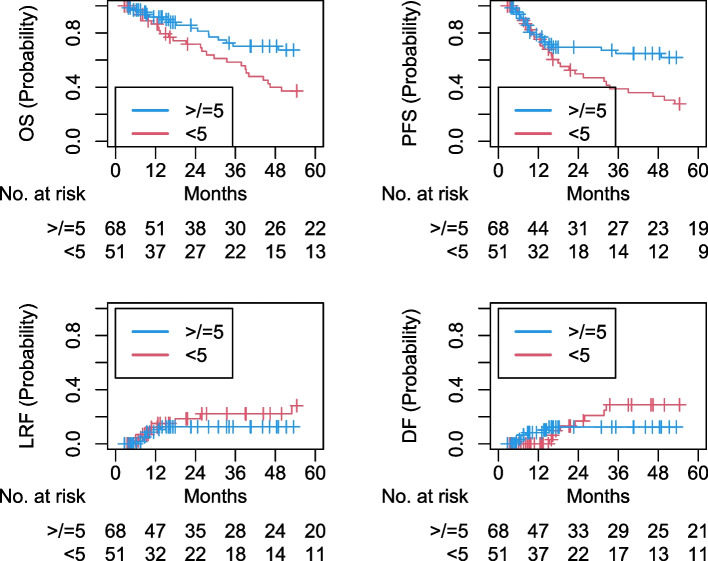
Kaplan–Meier and cumulative incidence curves for overall survival, progression-free survival, locoregional failure, and distant failure for greater than or equal to 5 versus fewer than 5 cycles of weekly low-dose cisplatin. OS: overall survival; PFS: progression-free survival; LRF: locoregional failure; DF: distant failure

On Cox MVA (Table 2), poor adherence to low-dose cisplatin was associated with worse OS (adjusted hazards ratio [aHR] 2.94, 95% confidence interval [CI] 1.58–5.47, *p* < 0.001) and PFS (aHR 2.32, 95% CI 1.29–4.17, *p* = 0.005; Fig. 1). On Fine-Gray MVA (Table 3), it was associated with worse DF (aHR 4.55, 95% CI 1.19–17.3, *p* = 0.03), but not LRF (aHR 1.61, 95% CI 0.46–5.58, *p* = 0.46; Fig. 1). On logistic MVA (Table 4), KPS < 90 was the only variable associated with poor adherence to low-dose cisplatin (adjusted odds ratio [aOR] 2.67, 95% CI 1.10–6.65, *p* = 0.03).

**Table 2:** Cox proportional hazards multivariable analysis for overall and progression-free survival outcomes

	Overall Survival			Progression-Free Survival		
	aHR	95% CI	P	aHR	95% CI	P
Cisplatin						
> / = 5 Cycles	Reference			Reference		
< 5 Cycles	2.94	1.58–5.47	< 0.001	2.32	1.29–4.17	0.005
Gender						
Male	Reference			Reference		
Female	0.76	0.31–1.87	0.55	0.42	0.15–1.18	0.1
Smoker						
Never	Reference			Reference		
Former	1.74	0.76–3.98	0.19	1.89	0.81–4.39	0.14
Current	1.61	0.58–4.49	0.36	1.38	0.51–3.75	0.53
Age						
< 65	Reference			Reference		
65 or older	0.85	0.46–1.58	0.62	0.73	0.40–1.33	0.3
KPS						
90–100	Reference			Reference		
< 90	1.93	1.02–3.66	0.04	1.91	1.04–3.50	0.04
Race						
White	Reference			Reference		
Other	0.94	0.38–2.33	0.89	1.14	0.48–2.70	0.76
BMI						
Normal	Reference			Reference		
Underweight	0.53	0.12–2.29	0.39	2.9	0.51–16.59	0.23
Overweight	0.52	0.26–1.05	0.07	0.78	0.39–1.56	0.48
Obese	0.88	0.38–2.08	0.78	1.13	0.51–2.49	0.77
Not available	< 0.001	< 0.001-Inf	1	< 0.001	< 0.001-Inf	1
Site						
Oropharynx	Reference			Reference		
Larynx	0.46	0.19–1.12	0.09	0.6	0.26–1.40	0.24
Oral cavity	1.91	0.17–21.13	0.6	Jan-84	0.17–20.13	0.62
Other	1.1	0.49–2.45	0.82	1.06	0.48–2.36	0.89
T staging						
1–2	Reference			Reference		
3–4	2.54	1.30–4.96	0.006	2.71	1.40–5.23	0.003
N staging						
0–1	Reference			Reference		
2–3	1.37	0.58–3.20	0.47	1.46	0.65–3.32	0.36
HPV						
Negative	Reference			Reference		
Positive	0.54	0.20–1.46	0.23	0.76	0.29–1.97	0.57
Not available	0.92	0.38–2.23	0.86	1.31	0.57–3.03	0.52

*aHR* Adjusted hazards ratio, *95% CI* *95%* Confidence interval, *BMI* Body mass index, *KPS* Karnofsky performance status, *HPV* Human papillomavirus

**Table 3:** Fine-Gray multivariable analysis for locoregional and distant failure outcomes

	Locoregional Failure			Distant Failure		
	aHR	95% CI	P	aHR	95% CI	P
Cisplatin						
> / = 5 Cycles	Reference			Reference		
< 5 Cycles	1.61	0.46–5.58	0.46	4.55	1.19–17.3	0.03
Gender						
Male	Reference			Reference		
Female	0.12	0.02–0.73	0.02	< 0.001	< 0.001- < 0.001	< 0.001
Smoker						
Never	Reference			Reference		
Former	6.38	0.24–170.72	0.27	5.69	0.57–57.1	0.14
Current	0.78	0.04–14.22	0.87	3.34	0.34–32.9	0.3
Age						
< 65	Reference			Reference		
65 or older	0.49	0.07–3.34	0.46	0.14	0.02–0.88	0.04
KPS						
90–100	Reference			Reference		
< 90	1.81	0.55–6.00	0.33	1.74	0.23–13.3	0.59
Race						
White	Reference			Reference		
Other	4.78	0.69–32.93	0.11	0.14	0.02–0.78	0.03
BMI						
Normal	Reference			Reference		
Underweight	64.4	7.90–524.98	< 0.001	< 0.001	< 0.001–0.001	< 0.001
Overweight	2.6	0.63–10.77	0.19	23	2.54–208	0.005
Obese	2.04	0.48–8.76	0.34	12.1	1.31–113	0.03
Not available	0.003	< 0.001–0.40	0.02	< 0.001	< 0.001–0.02	< 0.001
Site						
Oropharynx	Reference			Reference		
Larynx	0.98	0.15–6.52	0.99	2.75	0.27–28.2	0.39
Oral cavity	< 0.001	< 0.001–0.12	0.01	1500	22.2–1.01e5	< 0.001
Other	1.53	0.20–11.87	0.68	1.78	0.15–20.8	0.65
T staging						
1–2	Reference			Reference		
3–4	18.7	1.80–194.53	0.01	5.33	1.20–23.7	0.03
N staging						
0–1	Reference			Reference		
2–3	2.34	0.28–20.00	0.44	19.7	3.64–106	< 0.001
HPV						
Negative	Reference			Reference		
Positive	2.18	0.13–36.41	0.59	1.1	0.20–5.95	0.91
Not available	5.39	0.33–88.75	0.24	0.5	0.07–3.57	0.49

*aHR* Adjusted hazards ratio, *95% CI* 95% Confidence interval, *BMI* Body mass index, *KPS* Karnofsky performance status, *HPV* Human papillomavirus

**Table 4:** Logistic multivariable analysis for poor adherence to weekly low-dose cisplatin

	aOR	95% CI	P
Gender			
Male	Reference		
Female	0.92	0.25–3.16	0.9
Smoker			
Never	Reference		
Former	1.70	0.59–5.17	0.34
Current	3.25	0.81–14.09	0.1
Age			
< 65	Reference		
65 or older	1.27	0.53–3.07	0.59
KPS			
90–100	Reference		
< 90	2.67	1.10–6.65	0.03
Race			
White	Reference		
Other	3.09	0.92–11.56	0.08
BMI			
Normal	Reference		
Underweight	6.89	0.43–218	0.2
Overweight	1.7	0.58–5.23	0.34
Obese	1.7	0.49–6.25	0.41
Not available	< 0.001	NA-1.63e122	0.99
Site			
Oropharynx	Reference		
Larynx	1.63	0.48–5.61	0.43
Oral cavity	2.04e7	< 0.001-NA	0.99
Other	1.09	0.32–3.61	0.88
T staging			
1–2	Reference		
3–4	1.22	0.48–3.06	0.67
N staging			
0–1	Reference		
2–3	0.72	0.22–2.34	0.58
HPV			
Negative	Reference		
Positive	1.88	0.48–8.19	0.38
Not available	1.01	0.30–3.56	0.98

*aOR* Adjusted odds ratio, *95% CI* 95% Confidence interval, *BMI* Body mass index, *KPS* Karnofsky performance status, *HPV* Human papillomavirus

## Discussion

To our knowledge, this is the largest study of US head and neck cancer patients treated with definitive chemoradiation comparing tumor control and survival outcomes based on the level of adherence to weekly cisplatin. It suggested that over 40% of patients underwent fewer than 5 weekly cisplatin cycles and that low adherence to weekly cisplatin was an independent, adverse prognostic factor for worse survival and distant failure outcomes. Those with reduced adherence to weekly cisplatin were more likely to have poor performance status.

The NCCN guideline [1] and ongoing NRG HN 009 clinical trial protocol (ClinicalTrials.gov Identifier: NCT05050162) do not currently specify the minimum number of weekly cisplatin cycles to be given. Over 40% of patients in our study received less than 5 weekly cisplatin cycles. Such proportion of patients with low adherence to weekly cisplatin was higher than 13–32% reported in the literature for head and nek cancer [4, 6, 7] as well as cervical cancer [8, 9]. This discrepancy may be due to older age in our study with median 63.5 years and more than a quarter of patients with > 70 years of age. Other studies included patients with mean or median age younger than 60 years [4, 6, 7], so it may be possible that our study included more frail patient population.

Our study also suggested that low adherence to weekly cisplatin was associated with worse survival and distant metastasis outcomes, but not locoregional control. Our findings in survival outcomes are consistent with studies from Australia and India suggesting survival benefits associated with better adherence to weekly cisplatin [7, 17]. However, while the Australian study did not compare LRF and DF based on the level of adherence to cisplatin, our findings on locoregional control are not consistent with the Indian study suggesting worse LRF associated with poor adherence to cisplatin [7, 17]. Such discrepancy may be in part due to differences in patient populations. For instance, nearly half of patients in our study had p16-positive oropharyngeal cancer, while more than a third of patients in the Indian study had hypopharyngeal cancer, which has been known to have poor prognosis [18, 19].

Limitations of our study include a relatively small sample size of 119 patients, which may be due to the national guideline recommending high-dose cisplatin given every 3 weeks instead as a preferred, category 1 option [1]. As a result, performing subgroup analysis stratified by p16 status was not feasible in our study. Follow up period in our study was also short with median 19.8 months. The number of events, such as death and distant metastasis, were still sufficient to reach statistical significance, highlighting the importance of improved adherence to systemic therapies to prevent early recurrences. Our database also did not fully capture toxicity profiles or medical comorbidities, which may be additional reasons for poor adherence to weekly cisplatin. Other pertinent variables, such as alcohol consumption, were unavailable for analysis,

## Conclusion

Our study suggested that low adherence to weekly cisplatin defined as fewer than 5 weekly cycles can be seen in more than 40% of patients. Such poor adherence was an independent, adverse prognostic factor for worse survival and distant metastasis outcomes. Patients with low adherence to cisplatin were more likely to have poor performance status. Further investigations are warranted to improve the adherence to chemotherapy and outcomes.

## Data Availability

The data cannot be shared publicly due to the privacy of patients who participated in the study. The data are available from the corresponding author upon reasonable request.
